# The Rheological Properties and Texture of Agar Gels with Canola Oil—Effect of Mixing Rate and Addition of Lecithin

**DOI:** 10.3390/gels8110738

**Published:** 2022-11-15

**Authors:** Ewa Jakubczyk, Anna Kamińska-Dwórznicka, Anna Kot

**Affiliations:** Department of Food Engineering and Process Management, Institute of Food Sciences, Warsaw University of Life Sciences, 02-776 Warsaw, Poland

**Keywords:** agar, gel, dynamic shear rheology, compression test, texture, TPA, acoustic properties, syneresis, gel stability

## Abstract

This study aimed to determine the effect of different mixing rates and the addition of lecithin on the rheological mechanical, and acoustic properties of agar gels with the addition of canola oil. The mixing rate of the agar–oil mixture was changed from 10,000 to 13,000 rpm. Additionally, agar gels with the addition of lecithin from 1 to 5% were prepared. The frequency sweep test was used (at 4 and 50 °C) within the linear viscoelastic region (LVR) in oscillatory measurement. The agar–oil mixture was cooled from 80 to 10 °C, enabling the obtainment of the gelling temperature. Texture profile analysis (TPA) and compression tests, as well as the acoustic emission method, were applied to analyse the texture of the gels. The syneresis and stability of gels during storage were also measure. The increase in mixing rate in the case of agar gel with canola oil causes an increase in the elastic component of materials as well hardness and gumminess. Also, samples prepared with the higher mixing rate have more uniform and stable structures, with small bubbles. The increase in the concentration of lecithin is ineffective due to the formation of gels with a weak matrix and low hardness, gumminess, and stability during storage.

## 1. Introduction

Gels may play an important role in the formation of many novel products. Many hydrocolloids are applied in production of gels. However, agar–agar is an effective gelling agent because it can form stable gels over a wide temperature range [1,2]. The gelling temperature of agar gels is close to 40 °C, but the melting temperature is about 90 °C [3]. Agar gels can be also used in the production of food for older adults with dysphagia. The application of agar microgels structures allows the user to modify the texture of food to reduce aspiration risk. Agar gels achieve a higher yield stress and elastic modulus than xanthan gum solutions. It indicates that agar gel is a suitable ingredient in the diet of consumers with swallowing difficulties [4]. Agar can be also used in the fabrication of a controlled Pickering emulsion. Emulsions stabilized by canola protein microgels with agar represented the most elastic solid-like and viscous character in comparison to products with xanthan and gelatin. Rezaee et al. [5] concluded that such a viscoelastic gel-like systems could find potential application as fat replacers in novel food products. Wang et al. [6] noted that soybean oil-in-water (O/W) emulsion–agar gel characterised the limitation of digestion of products due to the entrapment of oil droplets in the agar network. The gelation of this emulsion had delayed lipid hydrolysis. The structure, as well as texture, are important properties of gels, because the functionality of a gelled material is linked with its rheological behavior during processing and mastication [7,8]. The physicochemical, mechanical, and rheological properties of agar gels are important. The high values of elastic moduli at low concentration provide many crucial functions of the agar gel [9], especially in designing properties of products. The texture and rheology attributes can be analyzed based on the different measurement methods when the small and the large strain deformation are applied. All these methods provide different information of gels products. The dynamic shear rate rheology can characterize the viscoelasticity of gels, as well the as transition of their properties from sol to gel state. The large deformation strain methods (compression test, TPA) are recommend to obtain mechanical attributes because they are related to many sensory features evaluated by consumers [10]. Additionally, some texture attributes are predicted based on the acoustic emission information recorded during the deformation of dry or wet samples [11,12,13,14].

The texture and structure of hydrogels can be modified by using lecithin during the process of gel production. Lecithin is an amphiphilic mixture of phospholipids, which can improve the functionality and applicability of gels by the rearrangement of designed materials structure. Lecithin can play a role as a gelling agent and as a surfactant in the presence of required solvent [15]. Lecithin is also widely used as an emulsifier in the food industry. Additionally, lecithin is also as source of phospholipids, which are substances that are beneficial to health [16]. Wang et al. used different emulsifiers in oil-in-water emulsion–agar gels. Their results show that adding the emulsifier reduces the aqueous gel strength.

The texture and structure of gels can be also changed by the incorporation of gas bubbles to sol with the application of different stirring rates [9,17], time of mixing [13,18], addition of foaming agent [19], or injection of gas [2]. Aeration of gels may lead to rheological modifications that suit the requirements of consumer in developing and designing of novel gels [2].

Interesting example of creations of new structure and properties of gels is the entrapping of oil droplets in a gel network [20]. Recently, a novel strategy to transform liquid oils forms into three-dimensional structures by adding a gelling agent was observed. The improvement in food fat profile and the possibility to deliver many nutrients and bioactive compounds in food matrices determine their great potential [21]. This system can be used to design low-fat products and for the encapsulation of lipophilic ingredients [22]. Oil added in the preparation of gels can behave as an inactive or active filler in the creation of a material matrix. It means that the presence of oil droplets may lead to a decrease or increase in gel strength [20,23]. These types of gels can be used to deliver hydrophilic as well as hydrophobics ingredients in systems consisting of water, oil, and gelling agent [24].

Food supplements and nutraceuticals such as essential fatty acids, vitamins, and carotenoids can be delivered in the form of soft and hard gels capsules and chewables [25,26]. Currently, there has been increasing demand for supplying important nutrients and supplements of diet in an attractive and well-tasting form. This includes ingredients that consumers are reluctant to consume but that are important for nutritional reasons. In the formulation of gel with oils, the fat-soluble ingredients have to be stirred throughout the continuous water phase as small oil droplets [26,27]. The selection of proper emulsifier and gelling agents is an important factor in the production of gelled products containing oil droplets. The oil phase can be a medium used to incorporate many bioactive compounds in gelled products. However, the mixing in the oil phase requires complex production processes, which should lead to obtaining a stable product during the manufacturing process and storage [26]. Vellido-Perez et al. [21] analyzed the lipid oxidation and size of droplets of oil-gelled systems with curcumin produced with different operating parameters (different homogenization speeds). However, the current literature about the effect of preparation conditions on the texture and stability of gels with oil is still limited.

The aim of this study to evaluate the effect of the application of different mixing rates and the addition of lecithin on the modification and creation of the rheological, mechanical, and acoustic properties of agar gels with the addition of canola oil.

## 2. Results and Discussion

### 2.1. Rheological Properties of Gels with Oil

**Figure 1:**
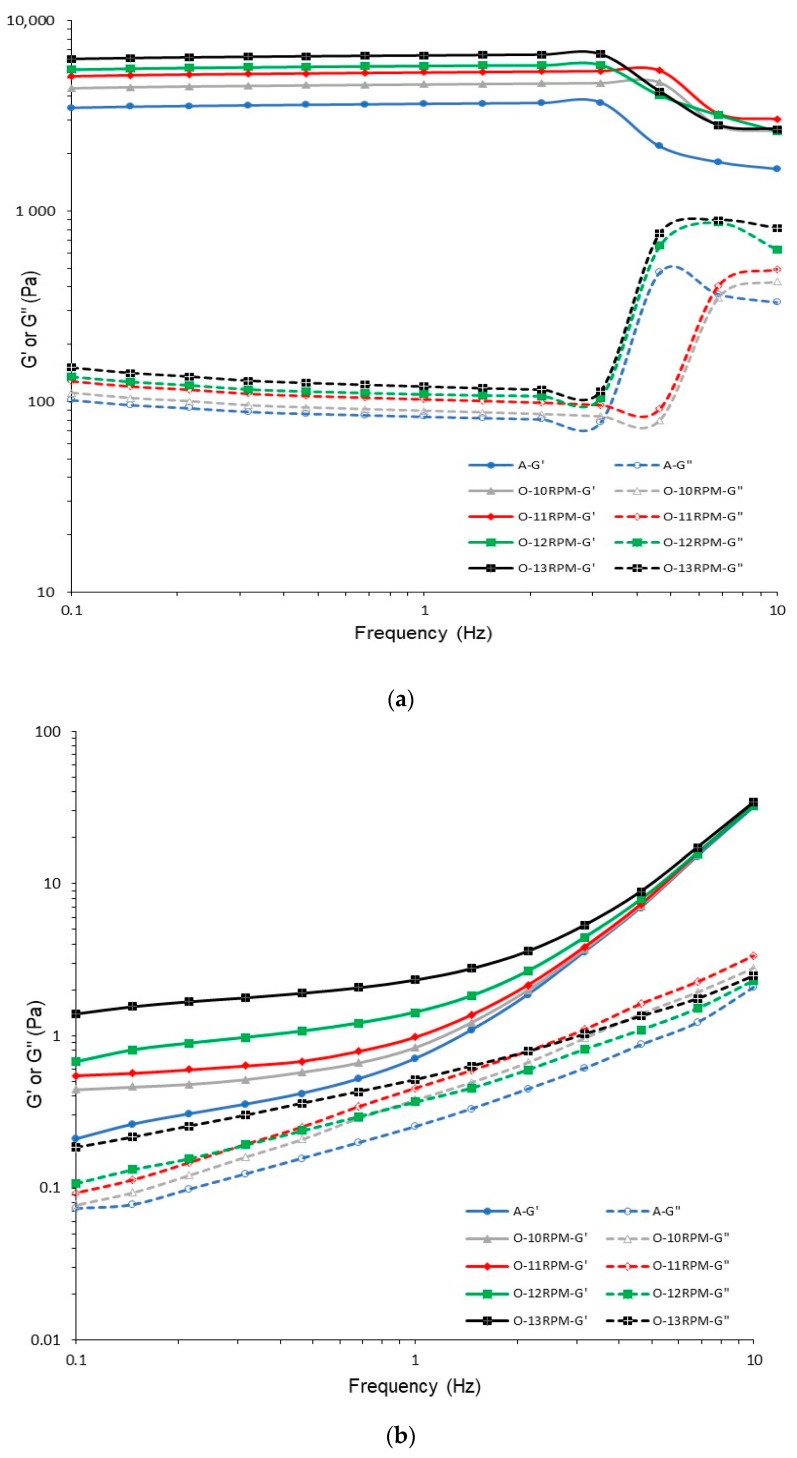
Frequency sweep curves of agar gel obtained with different mixing velocity at temperatures of 4 (**a**) and 50 °C (**b**).

The frequency sweeps of the of gels with oil and control agar gel (without oil) obtained with different mixing speeds during the homogenization of the water–oil mixture are presented in Figure 1. The rheological behaviour of samples was analyzed at temperatures of 4 and 50 °C. All samples show significantly higher values of G′ than G″ at the low temperature of 4 °C (Figure 1a). This behaviour is typical for the gel-like spectrum [22]. Additionally, slight changes in the values of G′ and G″ moduli with the frequency increase from 0.1 to 2 Hz are observed. The frequency independence is characteristic for a purely elastic gel [22,28]. The storage modulus (G′) develops similarly for all investigated samples at frequencies higher than 3 Hz (at a temperature of 4 °C). The decrease in G′ and the increase in G″ are observed at a frequency range from 3 to 10 Hz (Figure 1a). The significant increase in viscous component G″ can be linked with the failure of material structure and syneresis of gel (fluid leakage). The increase in mixing speed during the preparation of gels leads to increases in G′ and G″ values. It may indicate that fast-mixed samples are more elastic than non-mixed sample or material mixed at a low speed. This tendency is observed at temperatures of 4 and 50 °C (Figure 1a,b). Ross et al. [9] report that agar gels prepared with the fastest mixing speed have the highest values of complex modulus (at measurement temperature of 25 °C). Also, the large values of G′ in comparison to G″ are related to the elastic nature of agar gel obtained with slow (400 rpm) and fast mixing speeds (900 rpm). However, the index n of power low model describing the rheological behaviour of gels shows a lower value, indicating the more elastic properties of fast-mixed than non-mixed samples [9], which is in agreement with our results. Sun et al. [29] conclude that at the higher stirring speed, molecular chains have more chances of entanglement and a greater number of structures of aerogels are linked, which leads to the formation of a more organized matrix.

The application of higher temperature of frequency sweep test leads to a significant decrease in the G′ and G″ moduli (Figure 1b). The storage modulus has higher values than loss modulus in the whole range of applied frequency. Also, at the low frequencies, the G′ and G″ moduli are less dependent on the frequency. At frequencies higher than 1 Hz, a significant increase in the G′ and G″ moduli is observed. This phenomenon was described by Ghebremedhin et al. [30] in the case of agarose gels. The particle’s movement in these gels became faster with an increase in frequency. The particles were pushed together and interfered with each other at a higher frequency. The neighboring chains could collide and interfere, causing a higher increase in resistance with an increase in frequency. Figure 1a,b show that the effect of an increase in mixing speed on G′ and G″ moduli at 50 °C is similar as observed for samples measured at 4 °C. The fast-mixed samples have the higher values of storage and loss moduli than gels mixed at a lower speed.

**Figure 2:**
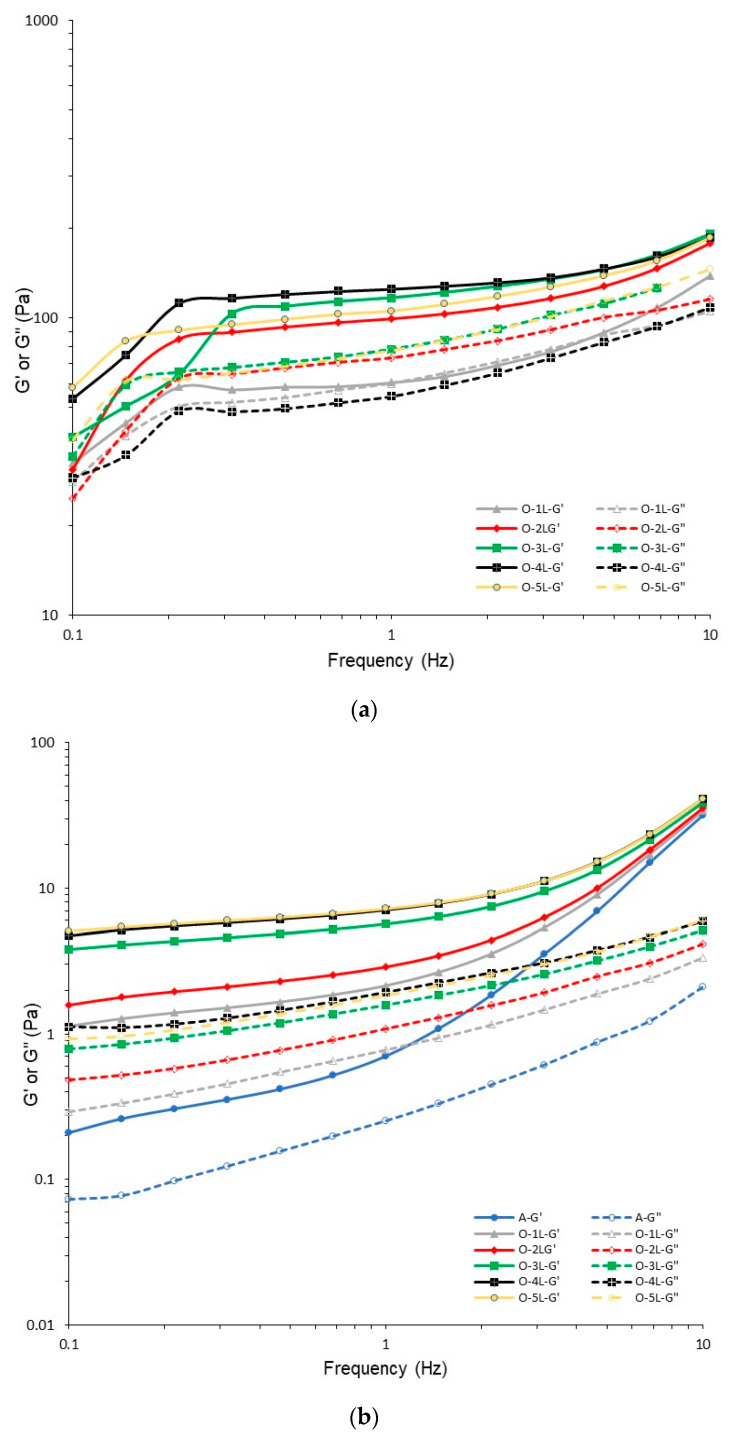
Frequency sweep curves of agar gel with different concentration of lecithin at temperatures of 4 (**a**) and 50 °C (**b**).

In the sample without oil (pure agar gel), the G′ and G″ are lower than for gels with 1% addition of oil. Zhao et al. [22] and Xi et al. [23] observe that incorporation of oil enhances the gel structure of emulsion gels. The oil can play an active (strengthen effect) or inactive (destructive effect) role in the formation of gel structure. However, the structure and strength of gel is dependent on the oil content [22,31], e.g., the samples with a low level of oil content show a decrease in G′ and G″ in starch–oil-based gels. Mhaske et al. [32] observed an increase in storage modulus G′ with the higher addition of canola oil in agarose gels. The enhancement in rigidity of the mixture is related to the increase in effective agarose concentration in the material (volume exclusion between aqueous and lipid phase). The addition of lecithin to agar gel prepared with addition of 1% of oil with the constant mixing rate causes a decrease in G′ and G″ moduli at a temperature of 4 °C (Figure 2a). The gels with oil obtained without addition of lecithin (O-10 rpm) and control agar gel (Figure 1a) are characterized by a high value of G′ modulus (4500 Pa) at a wide range of frequencies. Application of lecithin causes a decrease in storage moduli G′, to values lower than 200 Pa (Figure 2a). Also, the addition of Tween 20 as an emulsifier is found to reduce the G′ and G″ of emulsion gels with soya oil [33].

The values of the G′ and G′ moduli are dependent on the frequency of the whole range of this parameter. Many studies show that the changes in G′ with frequency increase is related to the viscoelasticity of materials. The significant decrease in gel strength (weaker structure of material) is linked with the greater dependence of G′ on the applied frequency [34,35,36]. Figure 2a shows that the values of the G′ modulus are higher than G″ for most oil–lecithin gels, with the exception of the sample with 1% of lecithin addition in the frequency range from 1 to 3 Hz. The results show that the addition of lecithin leads to the formation of a weaker product than oil gel prepared without phospholipid. Lecithin and water content play important roles in the formation of oleogels, because some studies show that below 7.5% lecithin, the gelation of the mixture is not observed at any level of added water. Bodennec et al. [37] confirm that a minimum concentration of lecithin (10%) is necessary to form oleogel structures that entrap oil. Also, an increase in lecithin concentration (up to 30%) leads to the formation of stable oleogels without phase separation during storage (3 months).

Ikeda and Foegeding [28] report that the protein gels without added lecithin characterizes the higher values of G′ for the mixed network when compared to a fine-strained network (lower concentration of NaCl). Additionally, the addition of lecithin causes the increase in the G′ of fine-strained networks, but a decrease in the particulate network. Heger et al. [15] note the addition of lecithin to agarose hydrogel increases the values of G′ and G″, which is correlated with the higher content of dry matter. Also, the storage modulus is dominant, which indicates the presence of gel with a fully cross-linked structure. The authors state that the presence of lecithin does not lead to a significant modification of the viscoelastic behaviour of agarose hydrogels in the range of lecithin concentrations from 0.5 to 2%. In our experiment, the addition of lecithin causes the opposite effect: the formation of gels with weak structures, which are probably not fully cross-linked. The interaction between oil–lecithin and agar has a crucial role in the formation of a weaker network of the gel.

Frequency sweep curves of agar gel with different additions of lecithin at a temperature of 50 °C show that the incorporation of phospholipid leads to an increase in the G′ and G′’ moduli (Figure 2b), which is in agreement with results obtained by Heger et al. [15] for agarose gels. Also, the increase in the concentration of lecithin causes the values of the viscoelastic parameters, which can be linked with the increase in dry matter content in agar–oil–lecithin mixtures.

**Figure 3:**
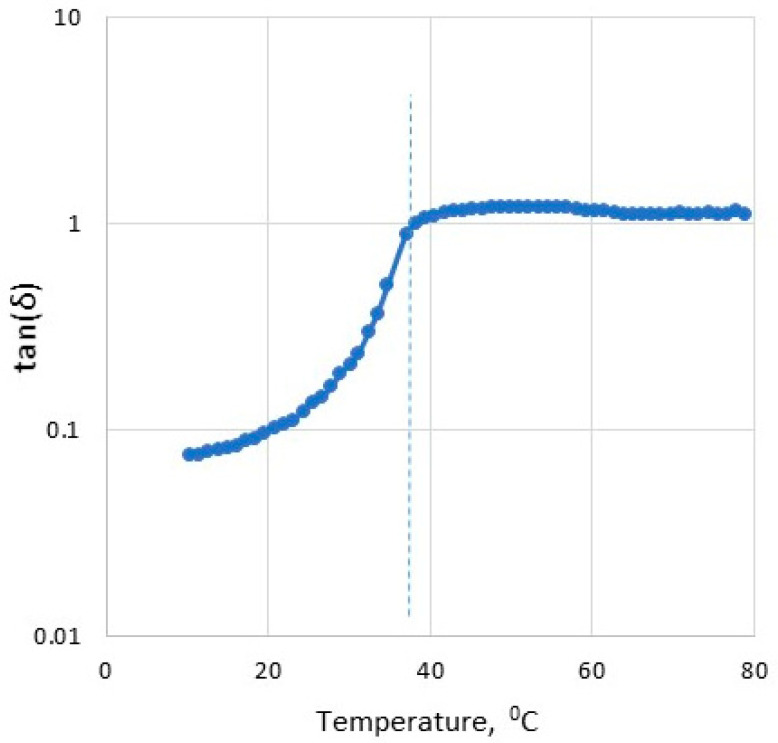
Tan (δ) as a function of temperature for agar during cooling of sample from 80 to 10 °C.

Figure 3 shows the changes of tan (δ) with the decrease in temperature during the cooling of the agar sol from 80 to 10 °C. The value of tan (δ) = 1 indicates the gelling temperature of agar (38.2 °C). The gelling temperature ranges from 34.4 to 38.2 for different variants of the investigated gels (Table 1). The similar range of initial gelling temperature was also obtained by Labropoulos et al. [38] for agar gel cooled at a different rate (34–37 °C). However, the increase in mixing speed, or the increase in lecithin concentration, does not affect the gelling temperature. The slightly higher gelling temperature is observed for pure agar gel (Table 1).

### 2.2. Texture of Gels with Oils

The results of the texture profile analysis (TPA) are presented in Table 2. The application of mixing and an increase in homogenization speed leads to obtaining oil–gels with a higher hardness and gumminess. Gumminess describes the amount of energy required to separate a semi-solid food sample to a state at which it can be swallowed [39]. The addition of oil also increases the hardness and gumminess of gels in comparison to pure agar gel. The results of small and large deformation tests of gels (dynamic shear rheology and TPA) indicate that the incorporation of oil and a higher speed of mixing may lead to the formation of a stronger structure of the gel with a higher strength. The springiness and cohesiveness of agar gel and samples prepared with oil and mixed at different speed do not differ, with the exception of gel prepared with the highest mixing rate (Table 2). The cohesiveness of this gel is slightly higher than observed for other samples. This texture attribute characterizes the toughness and the difficulty in destroying the product’s structure [39]. The effect of different mixing rates of pure agar gels on mechanical properties was investigated by Ross et al. [9]. The authors observed a higher elastic modulus for gel mixed at a faster rate. The fast mixing speed affects the degree of association between gels molecules and the formation of more aggregated double helices and suprahelices than is observed for slow mixing or not-mixed gels.

The addition of lecithin reduces the hardness and gumminess of gels with oils. The frequency sweep tests show that gels with the incorporation of lecithin form the weaker network of gels, which may also effect the texture of gels (Table 2). The values of springiness and cohesiveness are similar for gels with different concentration of lecithin.

The compression test confirms that the application of a fast mixing rate leads to an increase in the resistance of gels to deformation (Table 3). The fast-mixed gel has higher values of the compression work and Young’s modulus than samples prepared with a lower mixing speed, which is in agreement with the results obtained by Ross et al. [9].

The total acoustic energy and number of acoustic events were descriptors obtained based on the acoustic emission signal (Table 3). The composition and structure of samples affect the acoustic properties of deformed materials. The fractures of the network generated during the compression can be a source of acoustic signal containing information about texture properties of food samples [40]. The total acoustic energy, as well as the number of acoustic events, increases with mixing speed for agar gels. The agar gels prepared with the addition of foaming agents show the more intensive acoustic emission with the increase in foaming agent, which causes the increase in the number of bubbles of a smaller size [19]. The presence of air bubbles and oil droplets and their deformation during compression of gels may lead to the generation of cracks, and the failure of gels structures. These vibrations are a source of acoustic emission.

The different concentration of lecithin does not affect the compression descriptors of gels (Table 3). However, the compression work is characterized by significantly lower values than those obtained for gels with oils but without the addition of lecithin. The low mechanical strength also affects the acoustic properties of gels. The acoustic energy and number of events values are close to the acoustic background. The acoustic emission is not detected for samples with a higher addition of lecithin to gel.

The results of the texture tests, as well as acoustic emission measurement, confirm that the addition of lecithin (in the investigated range of concentrations) to gels with oil causes a worsening of their properties.

### 2.3. Stability and Structure of Gels

Gels made of cross-linked polymers such as polysaccharides store large amounts of solvent. The main problem with stability of these gel during the storage is their syneresis which related with the shrinkage of the gel matrix and the expulsion of solvent [41]. Syneresis index describes amount of solvent’s leakage from gels in comparison to initial mass of prepared gels. Table 4 shows that the syneresis is the highest for agar gel, and the addition of oil and the increase in mixing rate reduces the leakage of solvent from gel. Also, the increase in lecithin concentration does not affect the level of gel syneresis. The structure of gels with stronger bonds may retain the solvent in the gel network.

The TSI values are based on the transmission and backscattering over time. The increase in TSI indicates the occurrence of different phenomena such as creaming, flocculation, and coalescence in the material [42]. TSI values of gels after 7 days of storage show that the agar gel has the lowest stability over time (Table 4). The addition of oil and the increase in mixing speed lead to the decrease in TSI, which indicates the stable structure of material during the storage. The increase in lecithin concentration does not affect the TSI values for most samples.

**Figure 4:**
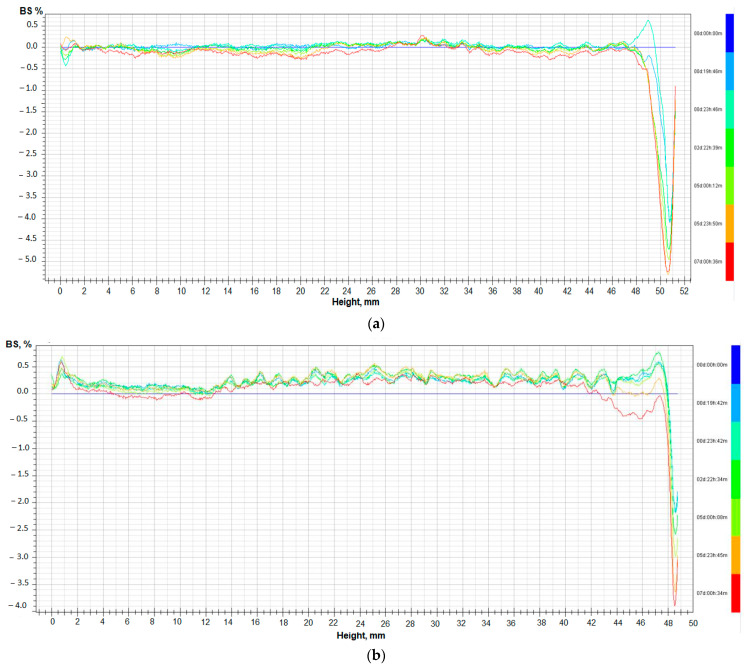
The variation of the delta backscattering of agar gel (**a**) and gel with oil and 1% of lecithin (**b**).

The results of back scattering (BS%) show that two different phenomena of destabilization occur in the case of agar gel and gel with lecithin (Figure 4a,b). Sedimentation, especially on the top of gels, and coalescence are observed for all gels with oil and for agar gel. Sedimentation is more visible in the agar gel sample, but coalescence is more intensive for samples with lecithin addition.

**Figure 5:**
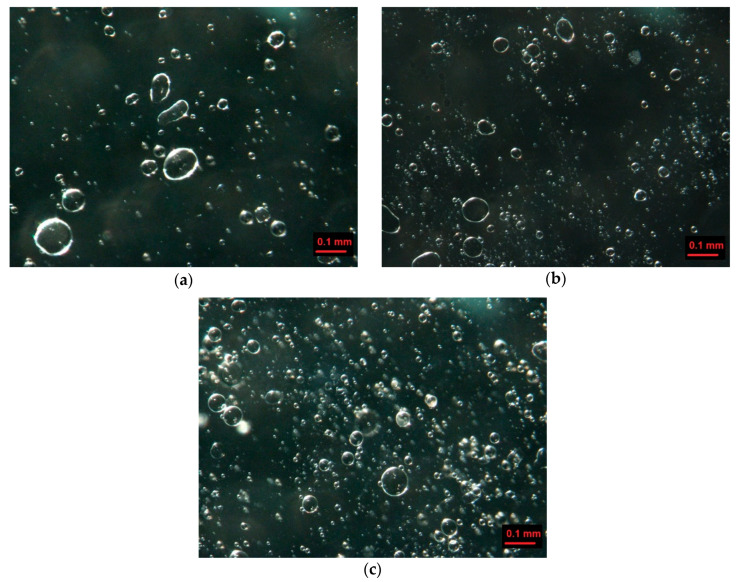
Structure of agar gel with oil prepared with different mixing rate: (**a**) 10,000, (**b**) 11,000, (**c**) 13,000 rpm.

Figure 5 shows examples of gels structures prepared with different mixing rates. The increase in mixing rate causes the decrease in the size of oil droplets and air bubbles. Additionally, the mixed gels with the highest speed contain a greater number of bubbles. Many studies [43,44] show that the increase in mixing rate generates a larger number of bubbles with smaller size, which is also observed for our study. The mean diameter of bubble and oil droplets decreases from 19 to 14 µm with an increase in the mixing rate from 10,000 to 11, 000 rpm. A further increase in speed to 12,000 and 13,000 rpm to leads to the production of bubbles with the mean diameter of 12 and 11 µm, respectively. The decrease in the size of droplets (with increase in mixing speed) causes the increase in G′ and G″. Matsumura et al. [33] observed that gels containing smaller oil droplets exhibit higher G′ and G″ values than corresponding gels with higher size of droplets.

The results of the texture measurements show that the most significant changes in many discriminants are observed with an increase in the mixing speed from 10,000 to 11,000 rpm. Hardness and compression work, as well as total acoustic energy and number of acoustic events, increase about 30–35% in this range of mixing rate. Wang et al. [6] observed that relative gel strength increased with the decrease in oil droplets in agar gels with soybean oil, which is consistent in our study.

The deformation of many smaller bubbles and droplets leads to the generation of a signal with a more intense acoustic emission. Also, the significant decrease in TSI values (about 25%) is characteristic for these gels (O-10RPM, O-11RPM). It shows that the stability of the gels increases with reduction in bubbles size and oil droplets. A further increase in speed to 12,000 and 13,000 rpm results in smaller changes in the values of most rheological, textural, and stability attributes. It may indicate that the structure of gels obtained at the highest mixing rates is similar. The structure of gels with different concentrations of lecithin do not differ and the mean size of bubbles is 22 µm. Also, many texture and stability descriptors obtained for gels with different additions of lecithin are similar.

## 3. Conclusions

The addition of oil and mixing of oil–agar sol with different speeds causes the increase in the elastic component of gels. This behaviour is observed for different temperatures of measurement (4, 50 °C). Gelling temperature is similar for all gels and ranges from 34.3 to 38.2 °C. A higher speed of mixing intensified the movement of molecules agar–oils phases, which accelerated the process of creating bonds and the formation of gel matrix. This leads to the formation of more uniform and organized structure with a higher number of smaller bubbles. The structure of these mixed gels with oil affects the texture of the product. The increase in the mixing speed results in the creation of gels with a higher hardness and gumminess, which leads to more intensive acoustic emission gels during the deformation. The values of lower values of syneresis index and TSI show that gel mixed quickly with oil has a more stable and stronger structure than agar gel or gels with addition of lecithin. Incorporation of lectin to agar gel with oil in concentration in the range from 1 to 5% is ineffective because the presence of this substance causes a reduction in the stability of gel, with the lowering of their elasticity and hardness. The application of mixing the high-speed oil is recommended to obtained stable gels with controlled textures.

## 4. Materials and Methods

### 4.1. Materials and Preparation of Gels

The samples of gels were prepared with the following ingredients: agar powder- Gelagar MU+ (Hortimex Sp. z o.o., Konin, Poland), canola oil (Edible Oils Limited, Szamotuły, Poland), lecithin (Sosa Ingredients S.L., Barcelona, Spain). The water with addition of 1% of agar powder were heated to 90 °C and stirred. The agar solution was cooled in the water bath until 50 °C and the canola oil was added (1% wt.). The mixture was homogenized at different rotation speeds (10,000, 11,000, 12,000, 13,000 rpm-samples: O-10RPM, O-11RPM, O-12RPM, O-13RPM) for 30 s using the IKA T25 Turrax dispenser (IKA-Werke GmbH & Co. KG, Oxford, UK). Additionally, agar sol and oil mixture were homogenized at constant speed 10 000 rpm with addition of different concentration of lecithin (1, 2, 3, 4, 5% wt., samples: O-1L, O-2L, O-3L, O-4L, O-5L). Lecithin was dispersed in oil and added to agar sol and then mixed. Additionally, the control sample of 1% of agar gel was prepared (with mixing and addition of lecithin).

### 4.2. Rheological Properties

Dynamic oscillatory shear tests were carried out using a controlled strain rheometer (a Haake Mars 40 rheometer, Thermo Scientific Inc., Karlsruhe, Germany) with a cup and bob geometry (CCB/CC25 DIN/Ti, with gap size 5.3 mm). The gel solution at temperature of 80 °C was added into the cup. To obtain the linear range for the dynamic analysis (LVR), strain sweep was conducted at 50 °C. Additionally, the LVR range was also analyzed after cooling the sample to temperature of 10 °C. Based on LVR results, the frequency sweep test was carried out at strain of 0.01 and at the frequency range from 0.1 to 10 Hz at temperature of 10 and 50 °C. The storage (G′) and loss moduli (G″), as well as tan (δ), were also measured during the temperature sweep. The sample was cooled at rate 2 °C·min^−1^ from 80 to 10 °C with 0.01 strain an at frequency of 1 Hz. All measurements were performed in triplicate for each type of gel material. Tan (δ) as a function of temperature for agar gel was analyzed to obtain the transition phase temperature from sol to gel (initial gelling temperature).

### 4.3. Mechanical and Acoustic Properties

The different agar mixtures with oil obtained with different rates of mixing, as well as with or/without lecithin, were poured on the Petri dishes and stored at 4 °C for 24 h. After storage, samples were diced into cubes with dimensions 12 × 12 × 12 mm.

The texture was tested with a TA-HD plus texture analyser (Stable Micro Systems, Surrey, UK). Tests were performed with a 20 mm flat-type probe. The measurements were recorded using the Texture Expert U.S. software. The following tests were performed:

- TPA at a sample deformation of 40% with a constant speed of 1 mm·s^−1^ and a pause between cycles of 5 s. Selected TPA parameters were described: hardness (N), springiness, cohesiveness, gumminess (N);
- Compression test with a sample deformation of 60% with speed of 1 mm·s^−1^; Compression work (mJ) (area under force–deformation curve) and Young’s modulus (kPa) (slope of a linear part of compression curve).

The acoustic emission descriptors were recorded during the compression test of sample gels. Acoustic emission signal (AE) was analyzed using a piezoelectric accelerometer type 4381 (Brüel and Kjær Naerum, Denmark). The number of AE events and total AE energy (arbitrary unit: a.u.) were obtained for investigated gels and analyzed according to the procedure applied by Jakubczyk et al. [13,14].

The mechanical and acoustic tests were carried out in 20 replicates for each variant of gel.

### 4.4. Syneresis and Stability of Gels

The syneresis test was carried out using a 4–15 SIGMA laboratory centrifuge with a 11,806 and 13,860 rotor. Sample of gels were placed in falcons (m_1_ = 5 g) and gels were centrifuged out for 10 min at room temperature, at a speed of 3700 rpm. Three tests were carried out for the tested gels. The supernatant was decanted and the mass of gel (m_2_) was measured. The parentage ratio of initial mass of gel m_1_ and the mass of remaining gels after centrifugation m_2_ was calculated as the syneresis index.

The stability of gels was recorded using Turbiscan Lab Expert (Formulation SA, Toulouse, France). Backscattered light (ΔBS%) was measured and Turbisoft 2.0.0.33 software enabled to calculate the Turbiscan Stability Index (TSI). This stability descriptor was measured during 7 days of the storage of gels at 4 °C. The measurements were made in triplicate.

### 4.5. Microstructure of Gels

The study was carried out using a stereoscopic SMZ 1500 microscope with a DS-Fi1 camera NIKON (Nikon, Shanghai, China). The sample of liquid gel was spread on a slide glass and gently covered using a coverslip. At least 5 photos for each type of gel were taken with application NIS-Elements BR 3.2. software (Nikon Instruments Inc., Melville, NY, USA). The substantial number of bubbles and droplets (N = 300) was measured to calculate their mean size diameter for all investigated gels.

### 4.6. Statistical Analysis

ANOVA test was applied to analyze the differences among samples at the 95% significance level. Significant pairwise differences were tested with application of Tukey’s test. The statistical analyses were performed with STATISTICA software v. 12.5. All the data were presented as the mean value ± standard deviation.

## Figures and Tables

**Table 1:** Gelation temperature (T_gel_) of different agar gels.

Kind of Gel	T_gel_ °C
A (0 RPM, 0 L)	38.2 ^Aa^
O-10RPM	35.8 ^b1^
O-11RPM	36.0 ^b^
O-12RPM	35.9 ^b^
O-13RPM	35.9 ^b^
O-1L	35.1 ^B2^
O-2L	35.0 ^B^
O-3L	34.7 ^B^
O-4L	34.3 ^B^
O-5L	34.4 ^B^

^1^ The different small letters indicate the significant difference between attributes in columns (effect of mixing speed—RPM). ^2^ The different capital letters indicate the significant difference between attributes in columns (effect of lecithin concentration—L).

**Table 2:** TPA parameters of different gels.

Kind of Gel	Hardness, N	Springiness	Cohesiveness	Gumminess, N
A	3.80 ± 0.20 ^dD^	0.90 ± 0.02 ^aE^	0.14 ± 0.01 ^bB^	0.52 ± 0.04 ^dC^
O-10RPM	4.08 ± 0.36 ^d^	0.92 ± 0.01 ^a^	0.13 ± 0.01 ^b^	0.54 ± 0.05 ^d^
O-11RPM	5.90 ± 0.64 ^c^	0.89 ± 0.03 ^a^	0.14 ± 0.02 ^b^	0.86 ± 0.16 ^c^
O-12RPM	7.08 ± 0.40 ^b^	0.88 ± 0.01 ^a^	0.20 ± 0.05 ^ab^	1.41 ± 0.04 ^b^
O-13RPM	8.46 ± 0.38 ^a1^	0.90 ± 0.02 ^a^	0.28 ± 0.04 ^a^	2.38 ± 0.48 ^a^
O-1L	1.29 ± 0.05 ^A2^	0.79 ± 0.05 ^A^	0.15 ± 0.01 ^B^	0.19 ± 0.01 ^A^
O-2L	1.00 ± 0.05 ^B^	0.83 ± 0.04 ^A^	0.19 ± 0.02 ^A^	0.19 ± 0.03 ^A^
O-3L	0.85 ± 0.1 ^C^	0.84 ± 0.05 ^AE^	0.19 ± 0.01 ^A^	0.16 ± 0.02 ^AB^
O-4L	0.85 ± 0.09 ^C^	0.85 ± 0.06 ^AE^	0.18 ± 0.01 ^A^	0.15 ± 0.01 ^B^
O-5L	0.72 ± 0.09 ^C^	0.83 ± 0.04 ^A^	0.18± 0.03 ^AB^	0.14 ± 0.02 ^B^

^1^ The different small letters indicate the significant difference between attributes in columns (effect of mixing speed: RPM). ^2^ The different capital letters indicate the significant difference between attributes in columns (effect of lecithin concentration: L).

**Table 3:** Selected mechanical and acoustic descriptors of gels.

Kind of Gels	Young’s Modulus, kPa	Compression Work, mJ	Total Acoustic Energy, a.u.	Number of Acoustic Events
A (0 RPM, 0L)	15.53 ± 1.18 ^bA^	4.6 ± 0.2 ^dC^	20 ± 4 ^bB^	5 ± 2 ^cA^
O-10RPM	16.53 ± 1.51 ^b^	6.2 ± 0.3 ^c^	22 ± 4 ^b^	8 ± 2 ^bc^
O-11RPM	18.83 ± 2.86 ^ab^	10.0 ± 1.1 ^b^	32 ± 6 ^a^	12 ± 3 ^ab^
O-12RPM	19.83 ± 2.07 ^ab^	12.4 ± 1.2 ^b^	38 ± 7 ^a^	14 ± 3 ^a^
O-13RPM	22.71 ± 1.86 ^a1^	15.7 ± 1.9 ^a^	45 ± 9 ^a^	17 ± 4 ^a^
O-1L	14.18 ± 1.00 ^A2^	1.0 ± 0.1 ^A^	7 ± 2 ^A^	3 ± 1 ^A^
O-2L	11.92 ± 1.94 ^AB^	0.8 ± 0.1 ^AB^	5 ± 2 ^A^	2 ± 1 ^A^
O-3L	11.50 ± 0.97 ^B^	0.7 ± 0.1 ^B^	nd ^3^	nd
O-4L	11.90 ± 1.25 ^B^	0.7 ± 0.1 ^B^	nd	nd
O-5L	10.72 ± 1.04 ^B^	0.6 ± 0.1 ^B^	nd	nd

^1^ The different small letters indicate the significant difference between attributes in columns (effect of mixing speed—RPM). ^2^ The different capital letters indicate the significant difference between attributes in columns (effect of lecithin concentration- L). ^3^ nd; the value was not detected.

**Table 4:** Syneresis index and TSI value of gels with oil.

Type of Gel	Syneresis Index, %	TSI
A	5.9 ± 0.7 ^aB^	5.12 ± 0.12 ^aC^
O-10RPM	3.8 ± 0.4 ^b^	0.52 ± 0.03 ^b^
O-11RPM	3.2 ± 0.4 ^bc^	0.39 ± 0.14b ^c^
O-12RPM	2.9 ± 0.3 ^c^	0.41 ± 0.01 ^c^
O-13RPM	2.7 ± 0.4 ^c1^	0.43 ± 0.07 ^c^
O-1L	4.3 ± 0.6 ^A2^	2.83 ± 0.57 ^A^
O-2L	4.2 ± 0.8 ^A^	1.91 ± 0.45 ^AB^
O-3L	4.0 ± 0.5 ^A^	1.81 ± 0.40 ^B^
O-4L	3.9 ± 0.7 ^A^	1.82 ± 0.70 ^B^
O-5L	3.9 ± 0.7 ^A^	1.81 ± 0.40 ^B^

^1^ The different small letter indicates the significant difference between attributes in columns (effect of mixing speed—RPM). ^2^ The different capital letter indicates the significant difference between attributes in columns (effect of lecithin concentration—L).

## Data Availability

Data obtained as described.
